# CircPCBL: Identification of Plant CircRNAs with a CNN-BiGRU-GLT Model

**DOI:** 10.3390/plants12081652

**Published:** 2023-04-14

**Authors:** Pengpeng Wu, Zhenjun Nie, Zhiqiang Huang, Xiaodan Zhang

**Affiliations:** 1 Anhui Province Key Laboratory of Smart Agricultural Technology and Equipment, Anhui Agricultural University, Hefei 230036, China; 2 School of Life Science, Anhui Agricultural University, Hefei 230036, China; 3 School of Information and Computer Science, Anhui Agricultural University, Hefei 230036, China

**Keywords:** plants, identify circRNAs, deep learning approach, raw sequences

## Abstract

Circular RNAs (circRNAs), which are produced post-splicing of pre-mRNAs, are strongly linked to the emergence of several tumor types. The initial stage in conducting follow-up studies involves identifying circRNAs. Currently, animals are the primary target of most established circRNA recognition technologies. However, the sequence features of plant circRNAs differ from those of animal circRNAs, making it impossible to detect plant circRNAs. For example, there are non-GT/AG splicing signals at circRNA junction sites and few reverse complementary sequences and repetitive elements in the flanking intron sequences of plant circRNAs. In addition, there have been few studies on circRNAs in plants, and thus it is urgent to create a plant-specific method for identifying circRNAs. In this study, we propose CircPCBL, a deep-learning approach that only uses raw sequences to distinguish between circRNAs found in plants and other lncRNAs. CircPCBL comprises two separate detectors: a CNN-BiGRU detector and a GLT detector. The CNN-BiGRU detector takes in the one-hot encoding of the RNA sequence as the input, while the GLT detector uses k-mer (k = 1 − 4) features. The output matrices of the two submodels are then concatenated and ultimately pass through a fully connected layer to produce the final output. To verify the generalization performance of the model, we evaluated CircPCBL using several datasets, and the results revealed that it had an F1 of 85.40% on the validation dataset composed of six different plants species and 85.88%, 75.87%, and 86.83% on the three cross-species independent test sets composed of *Cucumis sativus*, *Populus trichocarpa*, and *Gossypium raimondii*, respectively. With an accuracy of 90.9% and 90%, respectively, CircPCBL successfully predicted ten of the eleven circRNAs of experimentally reported *Poncirus trifoliata* and nine of the ten lncRNAs of rice on the real set. CircPCBL could potentially contribute to the identification of circRNAs in plants. In addition, it is remarkable that CircPCBL also achieved an average accuracy of 94.08% on the human datasets, which is also an excellent result, implying its potential application in animal datasets. Ultimately, CircPCBL is available as a web server, from which the data and source code can also be downloaded free of charge.

## 1. Introduction

A novel class of non-coding RNA is called circular RNA (circRNA). It is formed by a reverse connection of the downstream 5′ end splicing site and the upstream 3′ end splicing site, with a 3′,5′-phosphodiester bond at the junction. Post-splicing is another name for the procedure [1]. Since it lacks a free end, it was initially frequently dismissed as a by-product of incorrect splicing or operational errors and received little attention [2]. For the first time, viroids infectious to higher plants were covalently determined as circRNAs in 1976, according to Sanger et al. [3]. At this point, circRNAs began to attract widespread attention. CircRNAs have since been discovered in yeast mitochondria [4], the hepatitis delta virus (HDA) [5], humans [6], mice [7], and rats [8], among other places. There are many databases available now that can be used to accept and store circRNAs from various species, including circBase [9], circRNADb [10], PlantcircBase [11], etc. Compared with linear RNA, circRNA has a more stable and conserved closed-loop structure and is not degraded by RNA exonuclease enzymes.

**Figure 1:**
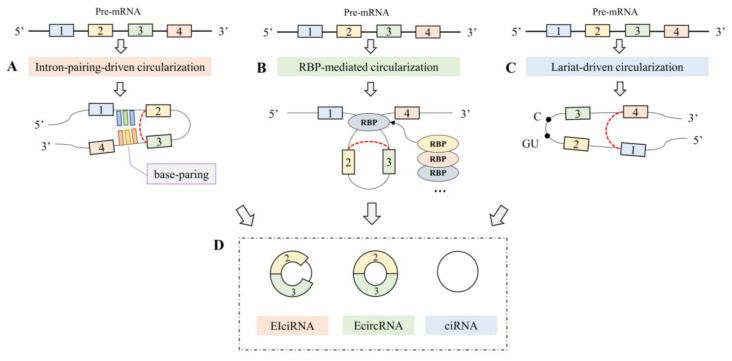
Biogenesis and structures of circRNAs: (**A**) intron-pairing-driven circularization; (**B**) RBP-mediated circularization; (**C**) lariat-driven circularization; (**D**) sdifferent circRNA structures. (EIciRNA: exon–intron circRNA; EcircRNA: exonic circRNA; ciRNA: intronic circRNA).

More and more circRNA functions are being annotated in the transcriptome as a result of the expansion of experimental techniques. For example, ciRS-7, which is abundantly expressed in human and mouse brains, acts as an miR-7 sponge and influences miRNA activity [12]. Li et al. found that intron-retaining circRNA could regulate the expression of the genes of RNA polymerase II [13]. CircRNA is critical for the emergence and growth of different cancer cells [14,15,16], according to recent research. Despite the fact that several functional circRNAs have been discovered, their formation mechanism is still not entirely understood. Intron-pairing-driven circularization, RNA-binding-protein (RBP)-mediated circularization, and lariat-driven circularization are the only patterns that have been observed thus far [2]. Figure 1 depicts these three mechanisms. In Intron-pairing-driven circularization, the intron sequences on both sides of the circularized exon can pair complementarily, permitting the direct combination of the 5′ splice site with the 3′ splice site, resulting in the formation of circRNAs. In RBP-mediated circularization, RBPs bind to specific motifs in the flanking intron sequences and facilitate tissue-specific circRNA formation. In Lariat-pairing-driven circularization, exons will occur while pre-mRNAs carry on GU/AG splicing, leading to lariat formation, which can execute reverse splicing and then form circRNAs. It is clear that there is still much to learn with circRNA research, which is still in its infancy.

LncRNAs are transcripts that are more than 200 nt in length and encode little or no protein. They can be categorized into different types based on the positioning of their coding sequences relative to protein-coding genes, such as sense, antisense, bidirectional, intronic, and intergenic lncRNAs. LncRNAs were initially considered to be transcriptional noise with no biological function, which were transcribed by RNA polymerase II [17]. Recent studies have shown the close association of lncRNAs with various diseases, and a large number of computational methods have been developed as a result [18,19,20,21]. LncRNAs are equally vital in plants, playing significant roles in various biological processes. For example, lncRNAs are essential in corresponding abiotic stresses in plants, as detailed by Li et al. [22], while Meng et al. identified 63 plant-growth-hormone-responsive lncRNAs [23]. In addition, silence lncRNA1459 and lncRNA1840 have been shown to delay the maturation of tomato plants [24]. It has been shown that lncRNA recognition models constructed on human datasets can be used for closely related vertebrates, but perform poorly on plant datasets [25], suggesting that there may be differences in lncRNA formation mechanisms and biological characteristics between plants and animals [26]. While various experiments have demonstrated that lncRNAs have essential functional properties, distinguishing them from mRNAs is challenging due to their shared features of a cap structure. Moreover, lncRNAs typically lack conserved sequences that can be used for detection, which drastically reduces the number of features available to the field of bioinformatics [27]. Our task is to classify circRNAs and lncRNAs, which will be more demanding than classifying circRNAs and mRNAs, as both of them are non-coding RNAs with more structural and functional similarities.

The initial stage of carrying out follow-up studies is the identification of circRNA. Traditional experimental methods are ineffective and require a lot of time and effort. Due to their comparable length distribution and low expression traits, circRNAs as a subclass of lncRNAs continue to be difficult to differentiate from other lncRNAs [28]. Currently, several computational methods have been developed to identify circRNAs. For example, the CirRNAPL [29] classifier adopts the extreme learning machine (ELM) method, which is refined by a particle swarm. Through CNN, DeepCirCode [30] detected circRNA reverse splicing sequences and outperformed conventional machine learning (SVM and RF). JEDI [31] introduces a cross-attention layer, which is superior to the existing tools in its effectiveness, to capture the deep interaction between the splicing points. Although some progress has been made in these methods, their application is mainly limited to human and mouse datasets, and datasets using plants have not been considered.

At present, JEDI is an outstanding predictor that was built for animal circRNAs in 2021 [31], which achieved a model accuracy of over 98% on human datasets and an accuracy of over 86% on cross-species testing on mouse datasets. These results demonstrate the excellent performance of the tool in identifying animal circRNAs. However, plant circRNAs differ from animal circRNAs in that they contain the following characteristics: (1) Repeating elements and reverse complementary sequences are less common in flanking introns [32]. (2) There are non-GT/AG splicing signals on both sides of circRNAs junction sites in rice, which is different from human circRNAs [33]. These variations indicate that the presence and use of circRNAs may differ between plants and animals. During our experiments, CircPCBL achieved a 94% accuracy or so on the validation set when trained on the human dataset alone, which was comparable to JEDI. However, the accuracy on the plant dataset was just above 85%, which further illustrates the above point. The research on circRNAs in plants is still in its early stages at the moment. Therefore, it is urgent to develop a plant-specific circRNA identification method to accelerate the research progress of plant circRNAs.

Consequently, in 2021, Yin et al. developed the plant-specific circRNA prediction software PCirc [34], which calculated k-mers, ORFs, and splicing junction sequences coding (SJSC) characteristics, which were predicted by a trained RF model. Specifically, the k-mer features represent the frequency of occurrence of neighboring k nucleotides, and a k-value range of 1–4 was chosen in this study. ORFs, which denote the protein-coding segment of a sequence, were used in PCirc as both ORF-coverage and ORF-length, referring to the proportion and length of the protein-coding region within the entire sequence. Additionally, SJSC is a vector comprising 50 bp sequences upstream and downstream of the splicing junction site, with each base represented by a corresponding numerical code. PCirc used rice as the training species and successfully predicted the circRNAs of the rice by the above three sets of features, and the average accuracy of a ten-times-ten-fold cross-validation reached over 99%. The software also demonstrated brilliant performance on the cross-species test, with an accuracy of 89.80% and 81.30% on Arabidopsis and maize datasets, respectively. Although this method has achieved excellent results, given that it only used rice as a training species, its level of universality is insufficient. Machine learning and deep learning methods require a large amount of data to better express the semantic information of circRNAs and lncRNAs for making robust predictions. Moreover, using machine learning methods requires manual feature extraction, which consumes plenty of time and effort [35]. Consequently, this study proposes a deep-learning-based model for plant circRNA identification, which learns features directly in the original sequence through the end-to-end features of the deep learning methods, thus avoiding the manual extraction of features required in machine learning methods. To fill the gap in the identification of plant circRNAs, it is necessary to uncover high-quality features based on the unique structure of plant circRNAs, whereas such specific features require a large amount of prior biological knowledge. By training the model on plant circRNAs, we anticipated that the deep learning would be able to automatically extract features that were specific to plants from the raw sequence data, distinguishing them from animal circRNAs. As a result, we thought about our study from the following perspectives: (1) expanding the number of species, (2) using deep learning to automatically extract features, and (3) discarding complex feature engineering, where the input to the model was only based on the original sequences.

In this study, we developed a depth recognition framework named CircPCBL based on the aforementioned factors. For the construction of the datasets, we selected six different training species, including *Arabidopsis thaliana*, *Brassica rapa*, *Zea mays*, *Oryza sativa Japonica*, *Solanum lycopersicum*, and *Solanum tuberosum*. After preprocessing, the datasets included 17,600 distinct circRNAs and lncRNAs, where circRNAs were coded as 1 and lncRNAs were coded as 0 in our task. These sequences were from different databases, namely PlantcircBase, CANTATAdb2.0, and GreeNC v1.12, which were detected with high confidence by bioinformatics tools or were experimentally validated with specific sequence information available from the databases. These data were divided according to a ratio of 7:3, of which 70% were used for the model training and 30% for the model validation and hyperparameter tuning. Furthermore, we constructed three independent test sets for three different plants (*Cucumis sativus*, *Populus trichocarpa*, and *Gossypium raimondii*), which were all from various families, as the training set for validating the cross-species prediction ability of the model. A total of 8739, 6611, and 4501 sequences were in the three test sets mentioned above, respectively, and the source of the test data was identical to that of the training and validation data. Similarly, we validated the utility of the model in the experimentally validated circRNAs and lncRNAs datasets as well, with 11 circRNAs in *Poncirus trifoliata* and 10 lncRNAs in rice. In terms of model architecture, CircPCBL consists of two parts: the CNN-BiGRU detector based on one-hot and the GLT detector based on k-mer. One-hot encodes the original sequence, and if the sequence length is less than a fixed value m, it will fill with zero-vectors at the end; otherwise, is directly truncated to m. The value of k in k-mer was set to 1–4 in our task, which was calculated from the original RNA sequences. One-hot and k-mer can represent sequences effectively and produce excellent outcomes in the majority of biological sequence recognition tasks without any prior biological knowledge [36,37,38,39]. Where k-mer can reflect the differences in sequence composition but cannot reflect the order of each base, one-hot makes up for this. Thus, the one-hot and k-mer characteristics were selected to enhance each other’s sequence information. These two features were based on the original sequence encoding only, as a one-hot-based encoding on pure sequences still only represents the sequences base by base and a k-mer approach simply adds oligonucleotides as potential sequence motifs. So, at this stage, there is no functionality or new functionality features given to identify circRNAs. Our ongoing research and development led to the creation of the CNN-BiGRU detector, which is also discussed in Section 2.1.1 and Section 2.1.2. To extract the local sequence information and decrease the model parameters and feature dimensions, GLT was introduced to the k-mer processing process. This architecture was inspired by an improved transformer model, DeLighT, which reduces parameter redundancy by introducing GLT, making the transformer deeper, faster, and stronger [40].

This paper makes the following contributions:

Only the original sequences were used for the feature extraction, avoiding complex feature engineering and paying less attention to the local region of the sequences.

A depth recognition framework named CircPCBL is proposed, which uses a CNN-BiGRU detector and a GLT detector, respectively, to process different features, rather than simply using a single model.

As far as we know, this is the first study to use a deep learning method in machine learning techniques to identify plant circRNAs and other lncRNAs.

CircPCBL also showed a brilliant generalization performance on plants from different families from the training verification set.

We provide an online web server for easy use: www.circpcbl.cn (accessed on 27 December 2022). The data and source code can also be downloaded for free through the web server.

## 2. Results

We assessed the robustness of CircPCBL in identifying plants’ lncRNAs and circRNAs by the following datasets: (1) Validation set of CircPCBL (2) Three independent test sets constructed for each of the three plants (*Cucumis sativus*, *Populus trichocarpa*, *Gossypium raimondii*) (3) Independent case study of *Poncirus trifoliata* and rice (Real Set). In this section, we will describe the evaluation strategies in order.

### 2.1. Performance of CircPCBL for Validation Sets

#### 2.1.1. Comparison of Traditional Deep Learning Methods and Coding Methods

In this section, we selected six traditional deep learning algorithms for comparison, namely RNN, BiRNN, GRU, BiGRU, LSTM, and BiLSTM, which are commonly used in the NLP field. We experimented with the word embedding and one-hot encoding methods in order to select the best approach. By using a sparse representation of various bases, one-hot is capable of reflecting the individual pieces of information in a sequence. Word embedding differs from one-hot in that it can capture the relationship among different bases and encode a sequence as a dense matrix. In our task, word embedding encoded a single base as a fifty-dimensional dense vector, whereas one-hot only encoded it as a four-dimensional binary vector. The comparison results (Table 1) showed that the BiGRU model encoded by one-hot performed the best, with four of the five metrics (accuracy: 0.8216, recall: 0.7992, F1: 0.8172, and MCC: 0.6438) being significantly higher than the other models. In addition, its precision (0.8360) was almost equal to the second-ranked model of word embedding—BiGRU (0.8370). Given that there were no significant differences between the two encoding methods, one-hot merely encoded a sequence as a four-dimensional sparse matrix in order to demonstrate robust performance, which greatly reduced the computing cost. Therefore, we decided to begin the model improvement with one-hot-BiGRU. To guarantee a fair comparison, we adjusted the number of hidden units in [20,30,40] for all the models to select the best parameter. The tuned number of hidden units for each model is shown in Table 1. Each model was trained with 200 epochs.

#### 2.1.2. The Effect of Hyperparameters on CNN-BiGRU’s Performance

**Figure 2:**
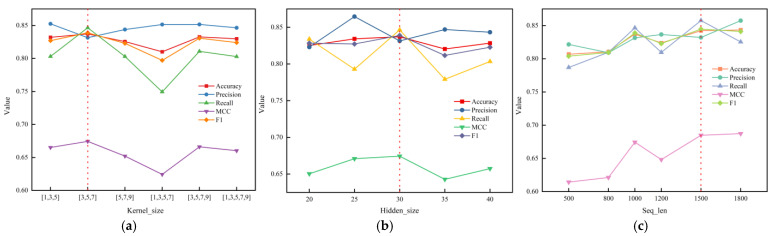
The effect of different hyperparameters on CNN-BiGRU: (**a**) Performance of CNN-BiGRU models with different kernel_size combinations; (**b**) effect of different hidden layer sizes on CNN-BiGRU models; (**c**) performance comparison of CNN-BiGRU models with different sequence fixed lengths.

To further improve the performance of the one-hot BiGRU model, we inserted CNNs before BiGRU to initially extract the local contextual information [41] and spatial information [42,43] of the sequences. CNN-BiGRU received one-hot encoding features as an input as well. In our experiments, CNN-BiGRU was proved to have a distinct performance improvement. We also examined the hyperparameters of CNN-BiGRU, such as the convolutional kernel size (Kernel_size), the number of hidden units (Hidden_size), and the sequence length (Seq_len), in order to improve the model’s performance. The results are shown in Figure 2.

The first hyperparameter was Kernel_size. We compared six combinations, including [1,3,5], [3,5,7], [5,7,9], [1,3,5,7], [3,5,7,9], and [1,3,5,7,9]. For each combination, we used 32 convolutional kernels to extract different features at the same scale. The combination of the convolution kernels with the best overall model performance was [3,5,7] (Figure 2a), where accuracy was 0.8371, precision was 0.8314, recall was 0.8465, F1 was 0.8389, and MCC was 0.6743. The overall model performance decreased when the combination of the convolution kernels was [1,3,5]. The problem was caused by the model’s perceptual field becoming narrower when a smaller convolutional kernel was used, making it impossible to capture the sequence’s overall contextual relationship. However, the model performance diminished to varying degrees as the convolutional kernels size or the number of kernels was increased. We speculate that, on the one hand, the addition of convolutional kernels increased the perceptual field and improved the ability to capture the global features of the sequence, but on the other hand, the expansion of the model parameters led to the expansion of the model parameters, which were easier to overfit and reduced the effectiveness of the model by increasing the amount of invalid information.

The second hyperparameter was Hidden_size. The representation of the semantic information included in the sequences depended on the size of the BiGRU hidden layer. Underfitting was more likely when there were too few hidden units present, while gradient disappearance was more likely when there were more hidden units present. In this regard, we experimented with every five values between twenty and forty as the hidden layer size. As shown in Figure 2b, when the number of hidden units was set to thirty, the four metrics of accuracy, recall, MCC, and F1 of the model reached the peak. Further increasing the hidden layer size did not improve the model’s performance but did increase the training time of the model. Thus, the Hidden_size hyperparameter was chosen to be 30 for the experiment.

The third hyperparameter was Seq_len. The amount of the sequence information maintained depended on the sequence’ fixed length size. It is clear from Figure 2c that there was a general positive correlation between the model’s performance and sequence length. The accuracy was poorer when the sequence length was short (500, 800) because the sequence lost too much information. Each measure had the following values when the sequence length was 1500: accuracy: 0.8422, precision: 0.8320, recall: 0.8576, MCC: 0.6848, and F1: 0.8446. The recall and F1 were greater by 0.0322 and 0.0036, respectively, as compared to the duration of 1800. Although accuracy, precision, and MCC were all greater for the constant length of 1800, there was not a significant difference overall. We ultimately decided on a value 1500 for Seq_len while also taking the computational cost into account.

Finally, we compared the overall model performance before and after the insertion of CNN (Table 2). The overall performance of CNN-BiGRU was better than that of BiGRU. Although the precision of CNN-BiGRU was lower, the gap between them was only 0.0039. For the training of the model, CNN-BiGRU was trained with 100 epochs, which was less than BiGRU, because it converged faster than BiGRU via our experimental findings.

#### 2.1.3. Performance after Fusion of the GLT Model

**Figure 3:**
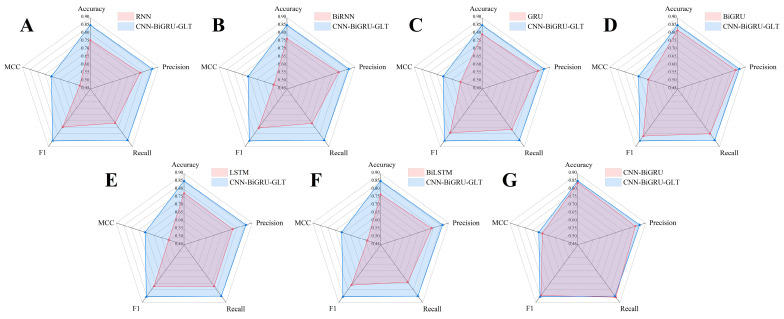
Comparison of the CNN-BiGRU-GLT model’s performance with traditional deep learning methods on validation set: (**A**) CNN-BiGRU-GLT and RNN performance comparison; (**B**) CNN-BiGRU-GLT and BiRNN performance comparison; (**C**) CNN-BiGRU-GLT and GRU performance comparison; (**D**) CNN-BiGRU-GLT and BiGRU performance comparison; (**E**) CNN-BiGRU-GLT and LSTM performance comparison; (**F**) CNN-BiGRU-GLT and BiLSTM performance comparison; (**G**) CNN-BiGRU-GLT and CNN-BiGRU performance comparison.

Finally, we improved CNN-BiGRU by fusing GLT to add additional sequence information. On the basis of the rule of just using raw sequences, we used k-mer features as the GLT model’s input. In theory, the deep neural network could learn directly from other sequence-based parameters such as GC content, purine, and pyrimidine content. The experimental comparison (Figure 3) showed that the model with GLT performed better than all the previous models, and, for the first time, the accuracy was greater than 85%. Accuracy, recall, MCC, and F1 were specifically enhanced by 0.0117, 0.0282, 0.0232, and 0.01, respectively, in comparison to CNN-BiGRU (Table 3). Therefore, we finally selected the CNN-BiGRU-GLT model as the plant circRNA and lncRNA recognition method.

**Figure 4:**
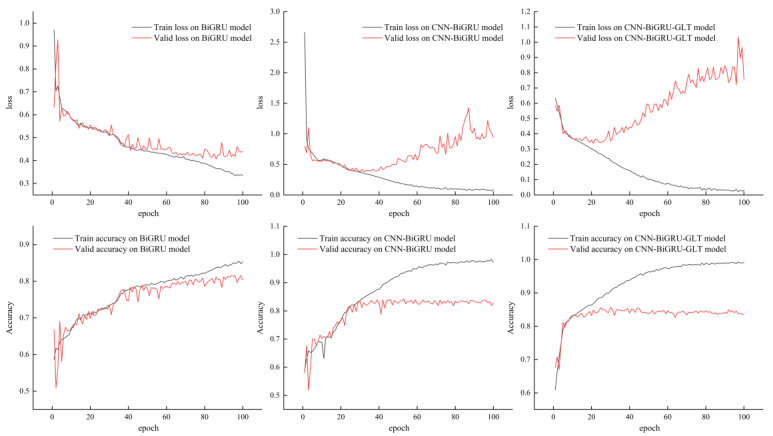
Loss and accuracy variation in each epoch for training and validation sets on different models with various degrees of improvement (BiGRU, CNN-BiGRU, and CNN-BiGRU-GLT).

We visualized the training process of the three models, BiGRU, CNN-BiGRU, and CNN-BiGRU-GLT, by plotting the changes in the loss and accuracy of the training and validation sets for the first 100 epochs, as shown in Figure 4. From the figure, it can be seen that the enhanced models converged faster and achieved higher accuracy rates.

In addition, we observed that the CNN-BiGRU-GLT model improved the accuracy by only around 1.2% compared to CNN-BiGRU, which may be attributed to the model stability factor. To further validate the model refinement, we repeated training the above two models five times, and the results (Table 4 and Table 5) showed that the CNN-BiGRU-GLT model outperformed it by consistently exceeding an 85% accuracy across all five experiments, while the CNN-BiGRU maintained an accuracy below 85%. In particular, the CNN-BiGRU-GLT model exhibited exceptional consistency, as all of its metrics maintained a standard deviation of less than 0.007. This is a testament to the model’s overall stability and reliability.

#### 2.1.4. Comparison of Traditional Machine Learning Methods

**Figure 5:**
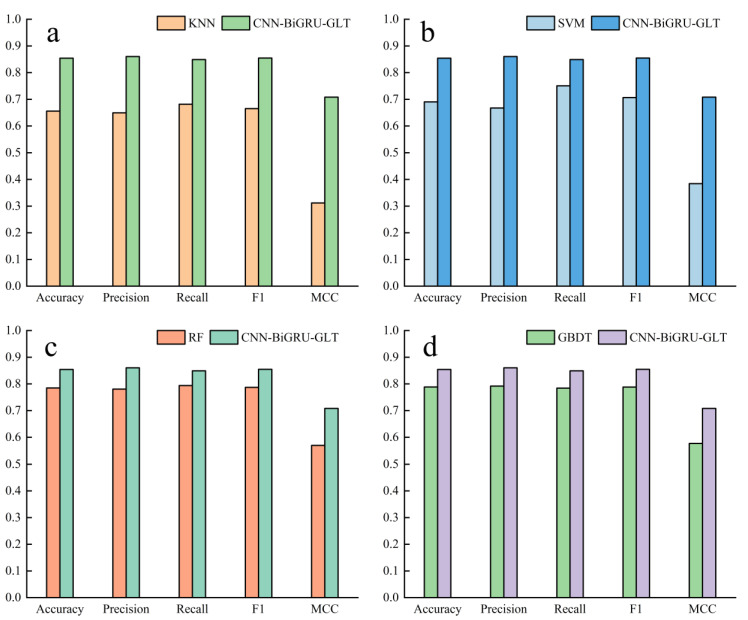
Comparison of the CNN-BiGRU-GLT model with traditional machine learning methods on validation set: (**a**) CNN-BiGRU-GLT and KNN performance comparison; (**b**) CNN-BiGRU-GLT and SVM performance comparison; (**c**) CNN-BiGRU-GLT and RF performance comparison; (**d**) CNN-BiGRU-GLT and GBDT performance comparison.

We also compared the performance of CircPCBL with four well-known machine learning algorithms (GBDT, RF, SVM, and KNN) to assess its performance more thoroughly. These machine learning methods utilized k-mer features as their inputs, with k being a value between 1 and 4. We tuned their hyperparameters by grid searching (details are shown in Table 6), and the adjusted parameters of each machine learning model were set as follows: GBDT {‘learning rate’: 0.1, ‘the number of base classifiers’: 200}; RF {‘the number of base classifiers’: 200}; SVM {‘kernel function’: Gaussian kernel function, ‘C’: 1.0}; KNN {‘the number of neighboring points’: 5, ‘*p*-value of Minsky distance’: 3}, and the rest of the parameters were taken as the defaults. The performances of the different machine learning models on the validation set are shown in Figure 5 and Table 7. We can see from the results that CircPCBL outperformed the more established machine learning techniques. In comparison to GBDT, RF, SVM, and KNN, the MCC value was 0.7080, which was 0.1311 higher, 0.1386 higher, 0.3239 higher, and 0.3964 higher, respectively. For the other metrics, accuracy was 0.0655, 0.0693, 0.1636, and 0.1983 higher than GBDT, RF, SVM, and KNN, respectively; precision was 0.0685, 0.0798, 0.1931, 0.2111 higher than GBDT, RF, SVM, and KNN, respectively; recall was 0.0647, 0.0553, 0.0985, and 0.1675 higher than GBDT, RF, SVM, and KNN, respectively; and F1 was 0.0666, 0.0676, 0.1482, and 0.1897 higher than GBDT, RF, SVM, and KNN, respectively. In addition, due to the generally low MCC values, it may be that the predictions were more accurate for a single class. As such, for a more robust assessment, we output the single-class prediction accuracy for each model. For circRNAs, CNN-BiGRU-GLT reached an accuracy of 0.8490, which was 0.0647, 0.0553, 0.0985, and 0.1675 higher than that of GBDT, RF, SVM, and KNN, respectively; regarding lncRNAs, CNN-BiGRU-GLT achieved an accuracy of 0.8590, which was 0.1312, 0.1386, 0.3274, and 0.3966 higher than that of GBDT, RF, SVM, and KNN, respectively. Therefore, it is evident that our model not only performed best in all the evaluation metrics but also yielded robust predictions for each category. At the same time, we noted that GBDT, the best-performing traditional machine learning algorithm, had an accuracy that was still somewhat inferior to that of BiGRU without any improvement, which indicated the effectiveness of the automatic feature extraction carried out by the deep learning methods.

### 2.2. Performance of CircPCBL for Test Sets

**Figure 6:**
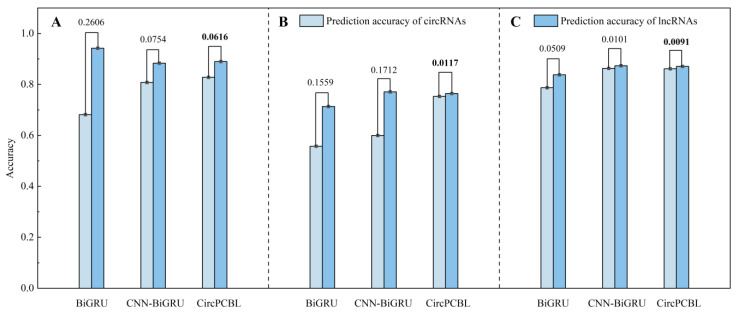
Single-class prediction accuracy with BiGRU, CNN-BiGRU, and CircPCBL models: (**A**) *Cucumis sativus*; (**B**) *Populus trichocarpa*; (**C**) *Gossypium raimondii*. The number on the bar chart indicates the prediction accuracy margin.

Similarly, we verified CircPCBL’s capacity for cross-species prediction and compared how well each model performed on the independent test set (Table 8). In this, RNN, BiRNN, GRU, BiGRU, LSTM, BiLSTM, and CNN-BiGRU had one-hot coding features as their inputs, the machine learning methods had k-mer features as their inputs, and CircPCBL had one-hot and k-mer features as its inputs. The results showed that *Cucumis sativus*, *Populus trichocarpa*, and *Gossypium raimondii* had prediction accuracies of 0.8588, 0.7587, and 0.8660, respectively. From the results, CircPCBL had the best performance across all measures and had a generalization ability that was much higher than that of the other models in terms of *Cucumis sativus* and *Populus trichocarpa*. The performance of the model in terms of *Gossypium raimondii* was not much lower than that of the top-ranked CNN-BiGRU in general. It is worth mentioning that the model’s generalization performance was significantly boosted with the addition of GLT, particularly on the independent test set for *Populus trichocarpa*. The model’s prediction accuracy on this test set was enhanced by almost 7% compared to its performance before incorporating GLT. Ultimately, to further illustrate the stability of the models and the effectiveness of the improvement strategy, we contrasted the models (BiGRU, CNN-BiGRU, and CNN-BiGRU-GLT) before and after their improvement via outputting their prediction accuracy for individual categories (Figure 6). The results showed that the CNN-BiGRU-GLT model accurately identified both circRNAs and lncRNAs on all the independent test sets, without any cases of superior identification for a specific class. Specifically, its difference in terms of the prediction accuracy for circRNAs and lncRNAs was the lowest with respect to the pre-improvement models BiGRU and CNN-BiGRU. In the *Cucumis sativus*, *Populus trichocarpa*, and *Gossypium raimondii* tests, the single-class prediction accuracy differences were 0.0616, 0.0117, and 0.0091, respectively, while these differences with the BiGRU model were 0.2606, 0.1559, and 0.0509, respectively, and those with the CNN-BiGRU model were 0.0754, 0.1712, and 0.0101, respectively. Meanwhile, it can be seen from Figure 6 that the BiGRU model had a clear case of biased prediction for the single-class test, and this situation was gradually corrected in the process of model refinement.

### 2.3. Prediction of Experimentally Validated circRNAs and lncRNAs

Zeng et al. identified 558 potential circRNAs in *Poncirus trifoliata* by high-throughput sequencing and bioinformatics analysis, and 11 circRNAs that were resistant to RNAse R were identified by real-time PCR [44]. The 11 circRNAs were subjected to CircPCBL, and a prediction accuracy of 90.9% was obtained, with 10 out of the 11 being correctly predicted as circRNAs. Other than the above, Li et al. used the rapid amplification of the cDNA ends method (RACE) to obtain ten lncRNA sequences that were present in rice [45]. These sequences were analyzed via the CircPCBL network as well, which successfully identified nine of them with an accuracy of 90%. These results indicate the usefulness of CircPCBL in identifying functional circRNAs and lncRNAs.

### 2.4. Smote Sampling for Different Species’ Sequences

The number of sequences remained different between the species, with *Arabidopsis thaliana* having the largest number of positive and negative samples of samples, each with a total of 3000. To ensure balanced datasets across all the species, we made use of smote sampling to increase the samples of the remaining five plants to six thousand. Table 9 displays the results obtained after retraining CircPCBL. The results showed that after smote sampling, the performance of CircPCBL slightly decreased on the validation set. Its biased prediction appeared more obvious on the three independent test sets, especially on the *Populus trichocarpa* test set, where its overall prediction accuracy reached 0.6719, but the prediction accuracy for circRNAs was only 0.5589. Smote sampling is theoretically a data enhancement method, but it did not show the desired effect in our task. We analyzed the possible reasons as follows.

Firstly, different species have vastly different sequence numbers. For example, the sequence number of *Brassica rapa* is merely 800, and expanding it to 6000 will not reveal much information, but it could increase the risk of overfitting in the model. Second, RNA sequences have structural and functional specificities. CircRNAs, as a subclass of lncRNAs, have a high similarity to them. Generating samples with a low volume based on the feature distance may produce more noise, which could adversely affect the learning process of the model. Eventually, the generated samples exhibited a high degree of similarity, which could cause the model to overly focus on these samples and result in overfitting, ultimately leading to a decrease in the generalization of the model.

### 2.5. Testing on Species of Wide Interest to the Field of Plant Genomics

**Figure 7:**
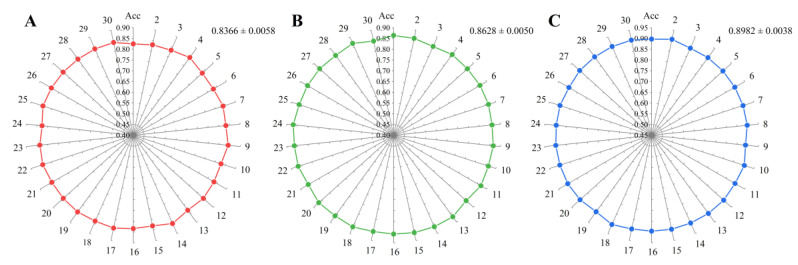
Thirty random sampling prediction experiments on plants of greater interest: (**A**) accuracy of each experiment on the *Arabidopsis thaliana* dataset; (**B**) accuracy of each experiment on the *Oryza sativa* dataset; (**C)** accuracy of each experiment on the *Solanum lycopersicum* dataset.

The species *Arabidopsis thaliana* [46], *Oryza sativa* [47], and *Solanum lycopersicum* [48] have been extensively studied in the field of plant genomics. To evaluate our model’s performance, we tested it specifically on these three species. In order to enhance the reliability of the prediction outcomes, we employed a random selection process to choose circRNA sequences from each species for analysis, where the numbers of *Arabidopsis thaliana*, *Oryza sativa*, and *Solanum lycopersicum* were 3000, 3000, and 2000, respectively. The process was repeated 30 times, and the final accuracy was the average result of these experiments. The results showed that the average prediction accuracy of *Arabidopsis thaliana* reached 0.8366 ± 0.0058, while that of *Oryza sativa* and *Solanum lycopersicum* was 0.8628 ± 0.0050 and 0.8982 ± 0.0038, respectively. The small variance of the 30 replicate experiments (Figure 7) not only demonstrates the robustness of the model but also proves that the randomly sampled data were representative of the overall data. To sum up, our software had an equally brilliant predictive power for the species of interest in the field of plant genomics.

### 2.6. Trying CircPCBL on Human Datasets

CircPCBL is a plant-specific tool, but we still tested its performance on a human dataset, hoping that the model could contribute to more species and not be limited to plants. Analogous to JEDI, our positive samples were from circRNADb (http://reprod.njmu.edu.cn/cgi-bin/circrnadb/circRNADb.php, accessed on 30 March 2023) [10], while the negative samples were from GENCODE v19 (https://www.gencodegenes.org/human/release_19.html, accessed on 30 March 2023) [49]. The numbers of positive and negative samples were 32,914 and 23,898, respectively. We randomly selected 8000 positive and negative samples of each, following a 7:3 division for training and validation. To guarantee the representativeness of the dataset, we repeated the process five times. For the model setup, 30 epochs were taken for the training, where an early stop was adopted to prevent overfitting, and the training time was approximately 24 min. The results (Table 10) showed that CircPCBL exhibited an average accuracy of 94.08% on the human dataset. Relative to positive-sample circRNAs, its average accuracy reached 94.31% without showing a biased prediction. Although the accuracy of CircPCBL was slightly lower compared to JEDI, it also achieved satisfactory results using only raw sequences to classify circRNAs and lncRNAs. While we did not carry out plant-specific feature engineering, CircPCBL showed large differences between the plant and animal datasets, which may imply that some variations existed in the plant and animal lncRNAs and circRNAs. In addition, when the CircPCBL model was trained on the plant dataset and directly transferred to the human dataset with the above five random samples (Table 10), its average accuracy was only 69.02%, and its F1 and MCC values were only 38.30%, and 67.10% on average, respectively. As a result, this also illustrates the necessity of developing plant-specific circRNA identification tools.

### 2.7. Impact of Training Species Diversity on Model Generalization Performance

In contrast to PCirc, we brought in more species for the training of the model. We argued that an increase in the number of species would improve the generalization performance of the model, for it could not only learn the similar aspects but also the different features via training on various kinds of plants. To verify this opinion, we applied only rice circRNAs and lncRNAs to CircPCBL and tested it on three independent test sets. The results (Table 11) showed that when trained only on rice, CircPCBL achieved accuracy, precision, recall, and MCC values of 0.8292, 0.8425, 0.8192, and 0.6586, respectively, on the validation set. However, the performance on the three independent test sets decreased remarkably compared to before, especially for *Cucumis sativus* and *Gossypium raimondii*, with the accuracy decreasing by 0.1612 and 0.1568, respectively. These results demonstrated that the number of species had an essential impact on the model’s generalization performance, and thus it is necessary to maintain the diversity of training species.

## 3. Discussion

The database PlantcircBase is devoted to cataloging the circRNAs of plants. It was created in 2017. Before that, almost all circRNAs databases were related to humans and animals [11]. Numerous studies on circRNAs in animals have been reported, but progress in studies on circRNA in plants has been sluggish. PlantcircBase has recently been updated to its seventh edition, which now includes a total of 171,118 circRNAs from 21 species of plants. Even though many plant circRNAs have been discovered, there are still no effective tools for identifying them, and only traditional experimental techniques are available. We believe CircPCBL to be the first deep-learning-based framework for circRNA identification in plants.

We used two different models and inputs in CircPCBL, and the outputs of the two models were linked for prediction through a fully connected layer. In particular, CNN-BiGRU was used to process the sparse matrix encoded by one-hot, and GLT was used to extract the deep-level information from the k-mer features. Different information about the sequences was processed independently using each of the two models. Through varieties of data testing and analysis, CircPCBL had an optimal stability and generalization. In addition, the improvement method was also shown to be effective for the CNN-BiGRU model. Our input did not consider any biological knowledge. We argue that biologically based characteristics (such as ORFs and CDs) focus excessively on the coding regions of RNA sequences and disregard the UTR regions, which leads to biased predictions for sequences with insufficient CD coverage [50]. CircPCBL lowers the attention on a single region by using one-hot and k-mer to reflect the sequence order and composition, respectively. It is also possible to fully display the composition information of the sequences. It is worth mentioning that CircPCBL, which is based only on the original sequence features, also showed brilliant performance under the different datasets.

However, CircPCBL might still require a lot of development. We plan to continue our research in the following areas: First, we will keep refining the model’s structure to enhance its prediction performance. Second, it has been found that the “tree” model in the machine learning algorithm has a better fitting ability, so we will consider the integration of the deep learning method and the “tree” model [51]. Third, we will explore high-quality features to facilitate our categorization task.

## 4. Materials and Methods

**Figure 8:**
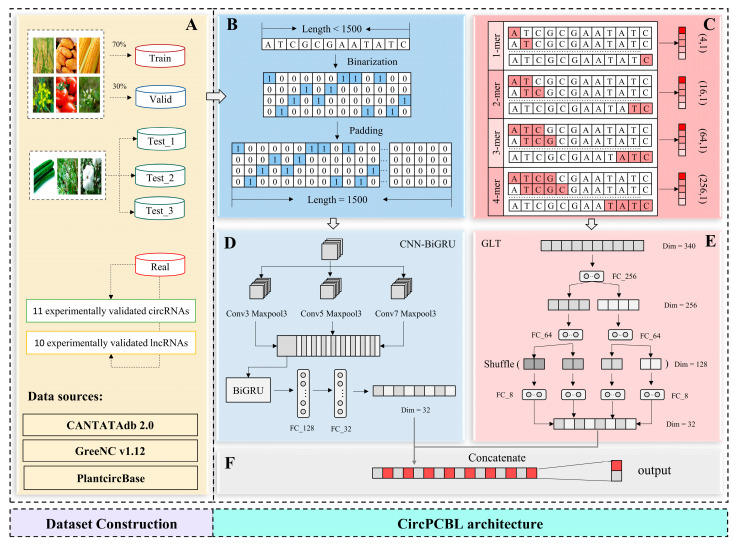
The flowchart for developing CircPCBL: (**A**) main datasets used in our work; (**B**) one-hot encoding process; (**C**) kK-mers features calculation process; (**D**) CNN-BiGRU model architecture; (**E**) GLT model architecture; (**F**) result output process.

The process of developing CircPCBL is shown in Figure 8. CircPCBL consists of two independent models (CNN-BiGRU and GLT) whose inputs are only based on the original sequences.

### 4.1. Dataset Construction

In this study, two classes were defined for the training of CircPCBL: lncRNAs from CANTATAdb 2.0 (http://yeti.amu.edu.pl/CANTATA/download.php, accessed on 27 December 2022) [52] and GreeNC v1.12 (http://greenc.sequentiabiotech.com/wiki/Main_Page, accessed on 27 December 2022) [53,54] were considered as the negative dataset; circRNAs, which were regarded as the positive dataset, were obtained from PlantcircBase (Release v7 Data) (http://ibi.zju.edu.cn/plantcircbase/, accessed on 27 December 2022) [11]. From all the above databases, we collected lncRNAs and circRNAs from nine different plants altogether. Among them, six types of plants (*Arabidopsis thaliana*, *Brassica rapa*, *Zea mays*, *Oryza sativa Japonica*, *Solanum lycopersicum*, and *Solanum tuberosum*) were used for constructing the training and validation sets, and they were divided according to a ratio of 7:3, and these plants belonged to the families Cruciferae, Gramineae, and Solanaceae. The remaining three types of plants (*Cucumis sativus*, *Populus trichocarpa*, and *Gossypium raimondii*) were constructed as three independent test sets, which do not belong to the same families as all the species in the training and validation sets. They were thus employed to confirm CircPCBL’s ability to predict the behavior across the species. Considering the problems of redundancy and imbalancing in the datasets, we performed the following processing on the raw data: Firstly, we removed the sequences with an excessively long or excessively short length from each fasta file using the box–whisker plot method. Next, we used the tools of cd-hit and cd-hit-est-2d with a threshold of 80% [55,56,57,58] to eliminate redundant sequences between the individual data sets and the different classes of datasets. Ultimately, we balanced the positive and negative samples for each species via random sampling. The specifics of the data used in our work are shown in Table 12. In addition to the above datasets, we also constructed a new test set, named the Real Set, which contained 11 circRNAs of *Poncirus trifoliata* reported in [44] and 10 lncRNAs of rice reported in [45] to further evaluate the generalization ability of CircPCBL under a real dataset [59,60]. Furthermore, we observed that there existed an imbalance in the datasets among the different species. To address this issue, after the final deployment of CircPCBL, we tried to perform smote sampling of sequences from various species, and we retrained the model to compare the before and after changes in the model’s performance.

### 4.2. CircPCBL Architecture

Deep learning has been used extensively in the field of biology recently [61,62,63,64,65]. End-to-end learning, which is possible with deep learning but not with traditional machine learning, reduces the amount of information needed to be understoon about circRNAs and lncRNAs and does away with the need for complex feature engineering [66]. Deep learning is a powerful tool for addressing high-dimensional datasets because it can automatically extract features from unprocessed sequences. Therefore, we will dig into the deep-level differences between circRNA and lncRNA by deep learning methods to realize the classification of circRNA and lncRNA.

After experimenting with different conventional deep learning models, we found that the model of BiGRU was more suitable for our classification task. By examining the performance of Word2Vec and one-hot, we finally decided to choose one-hot as the input of the BiGRU model. The model’s performance was then further enhanced by adding CNN in front of BiGRU. We also designed a GLT model, which received k-mer (k = 1, 2, 3, 4) features as its inputs in addition to CNN-BiGRU. K-mer was subjected to a grouped linear transformation via GLT to obtain local information, which was subsequently distributed via shuffling among many groups in order to acquire global representations. The outputs of the two models were connected, and the ultimate prediction results were output through the fully connected layer. Because gradient disappearance and overfitting are more likely to occur as a model’s depth increases, we used tactics such as early halting, layer normalization, and showing the learning rate. The details of the model are as follows.

#### 4.2.1. One-Hot CNN-BiGRU

One-hot encoding encodes four nucleotides as binary vectors, where: A(1,0,0,0), G(0,1,0,0), C(0,0,1,0), and T(0,0,0,1). Consequently, an RNA sequence of length L was represented by a 4xL sparse matrix. The length of the RNA sequences passed into the CNN-BiGRU model needed to be consistent, so we fixed the length of the sequences to m. Sequences with a length longer than m were truncated directly, and those with a length less than m were filled with (0,0,0,0,0) vectors. The CNN-BiGRU model calculated the output vector to have a 32-dimensional size for a single RNA sequence. We used ReLU for all activation functions. The sequence length was set to 1500. The convolution kernel size was a [3,5,7] combination, which we employed. The number of BiGRU hidden units was set to 30.

#### 4.2.2. K-mer GLT

The k-mer frequency is the frequency of the occurrence of adjacent k bases. It is thought that the k-mer frequency is species-specific and sequence-specific and that this specificity would become more pronounced as the k-value rises. Nevertheless, a dimensional catastrophe will result from the heedless pursuit of disparities in distribution [50]. We used 340 features, ranging from 1-mer to 4-mer, as the inputs into the GLT model. First, using a fully connected layer, we reduced the size of the features to 256 dimensions. Next, we divided the 256-dimensional vectors into 2 groups for linear transformation, with each group producing a 64-dimensional output vector. The 128-dimensional vector was finally divided into 4 groups for linear transformation, with the output vector’s dimensions being 8 for each group. This last step produced a 32-dimensional output vector. Layer normalization was used for each linear transformation to keep the gradient visible.

#### 4.2.3. Model Fusion

A fully connected layer then outputted the final prediction result after connecting the 32-dimensional vector that CNN-BiGRU and GLT both produced. In the whole training process, our learning rate was set to 0.001, and batch_size was set to 16. All the models were trained on an NVIDIA GeForce RTX 2060 GPU.

### 4.3. Performance Evaluation

To evaluate CircPCBL, we opted for some commonly used evaluation metrics, namely accuracy, precision, recall, F1-score, and MCC, which were calculated as shown below:

Accuracy = (TP + TN)/(TP + FP + TN + FN)(1)

Precision = TP/(TP + FP)(2)

Recall = TP/(TP + FN)(3)

F1-score = 2TP/(2TP + FP + FN)(4)

MCC = (TP × TN − FP × FN)/Sqrt((TP + FP) × (TP + FN) × (TN + FP) × (TN + FN))(5)

where TP and TN indicate the number of correctly predicted lncRNAs and circRNAs, and FP and FN indicate the number of incorrectly predicted circRNA and lncRNAs. Precision demonstrates how many of the predicted circRNAs samples were correct. Recall indicates how many circRNA samples were correctly predicted, i.e., the single-class prediction accuracy of circRNAs. The F1 score took into account both precision and recall, and its value is the harmonized average of them. MCC, whose full name is Matthew’s correlation coefficient, integrates TP, TN, FP, and FN, which can describe the correlation coefficient between the predicted and actual results. Its value ranged from −1 to 1, with higher values indicating better results for the model.

## 5. Conclusions

The abundance of plant circRNAs in PlantcircBase provides data support for deep learning techniques. The majority of circRNA recognition tools available today are geared toward animals, and it is still challenging to identify circRNA in plants. In this paper, we presented the CircPCBL model, which combines the models of CNN, BiGRU, and GLT and processes one-hot and k-mer features by different models for the identification of plant circRNAs. This model is based solely on raw sequences. CircPCBL has a wide range of species, is trained with various plant kinds, and exhibits outstanding cross-species prediction performance. In addition, we offer a free-to-use web server so that users can output predictions by simply entering the sequence as specified by the format or by directly uploading a fasta file. In a nutshell, CircPCBL is a user-friendly method for deeply identifying plant circRNAs and aims to increase the field’s understanding of these molecules. Despite the progress made with CircPCBL, there is still considerable scope for enhancing its accuracy, indicating the need for further development of similar tools. To improve the model’s performance, it would be useful to conduct error analyses to identify specific lncRNA subclasses or circRNA patterns that the model struggles to capture. This targeted information can inform future efforts to optimize the model’s performance, which is an area where our work can be further refined. We will explore how to further enhance the accuracy of plant circRNA prediction and conduct additional research on the functional prediction of plant circRNAs in a following study [67].

Lastly, since our tool classifies circRNAs and other lncRNAs, it cannot recognize other molecules such as mRNAs, tRNAs, etc. Here, we provide some suggestions to users to use our model better. Initially, in case you want to check the coding ability of sequences, we would recommend the following tools: OrfPredictor (http://proteomics.ysu.edu/tools/OrfPredictor.html, accessed on 6 April 2023) [68] or NCBI ORF Finder (https://www.ncbi.nlm.nih.gov/orffinder/, accessed on 6 April 2023) [69], and also LGC for long non-coding RNAs (https://ngdc.cncb.ac.cn/lgc/, accessed on 6 April 2023) [70]. In addition, when you are wondering if a sequence is a tRNA, please refer to the tool tRNA-scan (http://lowelab.ucsc.edu/tRNAscan-SE/, accessed on 6 April 2023) [71,72]. Each of these tools has proven to be powerful and user-friendly. Once you have verified that your test sequences are lncRNAs but not mRNAs or sncRNAs along with the above tools, our model will be of assistance in further determining whether they are circRNAs.

## Figures and Tables

**Table 1:** Performance of different deep learning models with different coding methods (the Time column indicates the model training time, and the Hidden column denotes the optimal number of neurons in the hidden layer for various models, with the range adjusted from [20,30,40]).

Code	Model	Hidden	Epochs	Time (min)	Validation Set				
					Accuracy	Precision	Recall	F1	MCC
One-hot	RNN	40	200	37	0.7729	0.7817	0.7590	0.7702	0.5461
	BiRNN	40	200	56	0.7771	0.7840	0.7665	0.7752	0.5543
	GRU	40	200	38	0.7784	0.8053	0.7359	0.7690	0.5590
	BiGRU	30	200	57	0.8216	0.8360	0.7992	0.8172	0.6438
	LSTM	20	200	38	0.6555	0.6769	0.5984	0.6353	0.3133
	BiLSTM	20	200	57	0.7763	0.7711	0.7877	0.7793	0.5528
Word embedding	RNN	30	200	38	0.7748	0.7771	0.7637	0.7703	0.5496
	BiRNN	20	200	57	0.7608	0.7848	0.7204	0.7512	0.5235
	GRU	30	200	39	0.7824	0.7833	0.7776	0.7804	0.5648
	BiGRU	30	200	59	0.8140	0.8370	0.7719	0.8031	0.6293
	LSTM	30	200	41	0.7752	0.7896	0.7630	0.7761	0.5508
	BiLSTM	30	200	61	0.7996	0.8187	0.7677	0.7924	0.6003

**Table 2:** Comparison of BiGRU and CNN-BiGRU performance on validation set.

Model	Epochs	Time (min)	Validation Set				
			Accuracy	Precision	Recall	MCC	F1
BiGRU	200	57	0.8216	0.8360	0.7992	0.6438	0.8172
CNN-BiGRU	100	46	0.8422	0.8320	0.8576	0.6848	0.8446

**Table 3:** Comparison of BiGRU and CNN-BiGRU performance on validation set.

Model	Epochs	Time (min)	Validation Set				
			Accuracy	Precision	Recall	MCC	F1
CNN-BiGRU	100	46	0.8422	0.8320	0.8576	0.6848	0.8446
CNN-BiGRU-GLT	100	88	0.8540	0.8603	0.8490	0.7080	0.8546

**Table 4:** Results of repeating the experiment five times for the CNN-BiGRU model.

CNN-BiGRU	Accuracy	Precision	Recall	MCC	F1
1	0.8422	0.8320	0.8576	0.6848	0.8446
2	0.8394	0.8704	0.7964	0.6812	0.8317
3	0.8422	0.8518	0.8296	0.6847	0.8406
4	0.8479	0.8439	0.8525	0.6959	0.8482
5	0.8445	0.8366	0.8590	0.6892	0.8477
Average	0.8433	0.8470	0.8390	0.6871	0.8426
Std	0.0032	0.0151	0.0266	0.0056	0.0068

**Table 5:** Results of repeating the experiment five times for the CNN-BiGRU-GLT model.

CNN-BiGRU-GLT	Accuracy	Precision	Recall	MCC	F1
1	0.8540	0.8603	0.8490	0.7080	0.8546
2	0.8570	0.8602	0.8534	0.7140	0.8568
3	0.8530	0.8574	0.8503	0.7061	0.8538
4	0.8557	0.8503	0.8620	0.7114	0.8561
5	0.8525	0.8534	0.8431	0.7048	0.8482
Average	0.8544	0.8563	0.8516	0.7089	0.8539
Std	0.0019	0.0044	0.0069	0.0038	0.0034

**Table 6:** Parameter tuning details of machine learning models (GBDT, RF, SVM, and KNN).

Model	Parameter	Description	Search Scope	Best
GBDT	learning_rate	Learning rate	[0.1, 0.01, 0.001]	0.1
	n_estimators	The number of base classifiers	[50, 100, 150, 200]	200
RF	n_estimators	The number of base classifiers	[50, 100, 150, 200]	200
SVM	kernel	Kernel function	[rbf, linear]	rbf
	C	Penalty factor	[0, 0.2, 0.4, 0.6, 0.8, 1.0]	1
KNN	n_neighbors	The number of neighboring points	[5, 10, 15]	5
	p	*p*-value of Minsky distance	[1, 2, 3]	3

**Table 7:** Specific metric values of the CNN-BiGRU-GLT model compared to traditional machine learning methods on the validation set.

Model	Time (min)	Validation Set				
		Accuracy	Precision	Recall	F1	MCC
CNN-BiGRU-GLT	88	0.8540	0.8603	0.8490	0.8546	0.7080
GBDT	3	0.7884	0.7918	0.7843	0.7880	0.5769
RF	<1	0.7847	0.7805	0.7937	0.7870	0.5694
SVM	<1	0.6903	0.6672	0.7505	0.7064	0.3842
KNN	<1	0.6557	0.6492	0.6815	0.6649	0.3117

**Table 8:** Performance of the CNN-BiGRU-GLT model on three independent test sets and comparison results with aforementioned traditional machine learning and deep learning methods.

Species	Model	Accuracy	Precision	Recall	MCC	F1
*C. sativus*	RNN	0.7508	0.6481	0.7500	0.4900	0.6952
	BiRNN	0.7659	0.6673	0.7628	0.5194	0.7118
	GRU	0.7574	0.6795	0.6816	0.4850	0.6805
	BiGRU	0.8119	0.7588	0.7386	0.5985	0.7485
	LSTM	0.7746	0.6967	0.7178	0.5241	0.7071
	BiLSTM	0.7687	0.6671	0.7784	0.5287	0.7185
	CNN-BiGRU	0.8457	0.7899	0.8080	0.6739	0.7989
	CNN-BiGRU-GLT	0.8588	0.8051	0.8280	0.7019	0.8164
	GBDT	0.7913	0.7183	0.7395	0.5593	0.7287
	RF	0.7911	0.7122	0.7531	0.5616	0.7321
	SVM	0.7120	0.6057	0.6882	0.4063	0.6443
	KNN	0.6295	0.5075	0.7652	0.3057	0.6103
*P. trichocarpa*	RNN	0.6600	0.6595	0.6495	0.3198	0.6545
	BiRNN	0.6218	0.6135	0.6415	0.2442	0.6272
	GRU	0.6618	0.6476	0.6974	0.3249	0.6716
	BiGRU	0.6356	0.6559	0.5577	0.2733	0.6028
	LSTM	0.6314	0.6188	0.6684	0.2640	0.6426
	BiLSTM	0.6507	0.6290	0.7206	0.3054	0.3054
	CNN-BiGRU	0.6854	0.7191	0.5998	0.3750	0.6540
	CNN-BiGRU-GLT	0.7587	0.7587	0.7529	0.5174	0.7558
	GBDT	0.6317	0.6115	0.7050	0.2673	0.6550
	RF	0.6226	0.5938	0.7563	0.2564	0.6652
	SVM	0.6303	0.5863	0.8639	0.2981	0.6986
	KNN	0.5624	0.5469	0.6852	0.1307	0.6083
*G. raimondii*	RNN	0.6874	0.4385	0.7775	0.3805	0.5607
	BiRNN	0.6636	0.4180	0.7922	0.3595	0.5472
	GRU	0.7345	0.4886	0.7455	0.4261	0.5903
	BiGRU	0.8125	0.6032	0.7870	0.5630	0.6829
	LSTM	0.7294	0.4825	0.7498	0.4211	0.5871
	BiLSTM	0.6992	0.4515	0.8026	0.4087	0.5779
	CNN-BiGRU	0.8683	0.6962	0.8632	0.6876	0.7708
	CNN-BiGRU-GLT	0.8660	0.6919	0.8615	0.6829	0.7675
	GBDT	0.7278	0.4804	0.7429	0.4156	0.5835
	RF	0.6943	0.4424	0.7351	0.3668	0.5524
	SVM	0.6485	0.4065	0.8035	0.3481	0.5398
	KNN	0.5279	0.3234	0.7688	0.1912	0.4553

**Table 9:** Performance comparison of CircPCBL on validation and independent test sets before and after SMOTE sampling.

Dataset	Model	Accuracy	Precision	Recall	MCC	F1
Validation set	CircPCBL	0.8540	0.8603	0.8490	0.7080	0.8546
	SMOTE + CircPCBL	0.8418	0.8434	0.8413	0.6835	0.8424
Test set_*C. sativus*	CircPCBL	0.8588	0.8051	0.8280	0.7019	0.8164
	SMOTE + CircPCBL	0.8479	0.8079	0.7857	0.6754	0.7966
Test set_*P. trichocarpa*	CircPCBL	0.7587	0.7587	0.7529	0.5174	0.7558
	SMOTE + CircPCBL	0.6719	0.7170	0.5589	0.3511	0.6282
Test set_*G. raimondii*	CircPCBL	0.8660	0.6919	0.8615	0.6829	0.7675
	SMOTE + CircPCBL	0.8660	0.7069	0.8165	0.6691	0.7577

**Table 10:** Performance of CircPCBL on human dataset.

Human Datasets	Accuracy	Precision	Recall	MCC	F1
After retraining	0.9408 ± 0.0025	0.9373 ± 0.0052	0.9431 ± 0.0106	0.8818 ± 0.0051	0.9401 ± 0.0029
Direct transferred	0.6902 ± 0.0012	0.7153 ± 0.0023	0.6320 ± 0.0055	0.3830 ± 0.0023	0.6710 ± 0.0025

**Table 11:** Performance of CircPCBL on three independent test sets when training with rice only (the values in parentheses show the decrease in contrast to previous tests).

Species	Accuracy	Precision	Recall	MCC	F1
*C. sativus*	0.6976 (−0.1612)	0.5895 (−0.2156)	0.6659 (−0.1621)	0.3753 (−0.3266)	0.6254 (−0.1910)
*P. trichocarpa*	0.6630 (−0.0957)	0.6556 (−0.1031)	0.6748 (−0.0781)	0.3262 (−0.1912)	0.6651 (−0.0907)
*G. raimondii*	0.7092 (−0.1568)	0.4554 (−0.2365)	0.6805 (−0.1810)	0.3589 (−0.3240)	0.5456 (−0.2219)

**Table 12:** Details of the datasets used in our work.

Dataset	Species	circRNA Code: 1			lncRNA Code: 0		
		Raw *	After **	Used ***	Raw *	After **	Used ***
Training and validation	*A. thaliana*	52,393	21,827	3000	4373	3324	3000
	*B. rapa*	591	480	400	8501	6473	400
	*Z. mays*	10,381	6262	600	10,761	682	600
	*O. sativa*	43,883	25,230	2000	2788	2088	2000
	*S. lycopersicum*	3796	2457	2000	4716	3124	2000
	*S. tuberosum*	1728	805	800	5790	3488	800
	Total	112,772	57,061	8800	36,929	19,179	8800
Test	*C. sativus*	4832	3313	3313	7348	5426	5426
	*P. trichocarpa*	4408	3278	3278	4322	3333	3333
	*G. raimondii*	1478	1155	1155	4216	3346	3346

* Raw data; ** data after redundant sequences were removed; *** balanced data for positive and negative samples (test datasets did not conduct the operation).

## Data Availability

The data and source code presented in this study are available at https://github.com/Peg-Wu/CircPCBL, accessed on 6 April 2023.
